# Melatonin Modulates Plant Tolerance to Heavy Metal Stress: Morphological Responses to Molecular Mechanisms

**DOI:** 10.3390/ijms222111445

**Published:** 2021-10-23

**Authors:** Md. Najmol Hoque, Md. Tahjib-Ul-Arif, Afsana Hannan, Naima Sultana, Shirin Akhter, Md. Hasanuzzaman, Fahmida Akter, Md. Sazzad Hossain, Md. Abu Sayed, Md. Toufiq Hasan, Milan Skalicky, Xiangnan Li, Marián Brestič

**Affiliations:** 1 Department of Biochemistry and Molecular Biology, Khulna Agricultural University, Khulna 9100, Bangladesh; najmulhaque466@gmail.com; 2 Department of Biochemistry and Molecular Biology, Bangladesh Agricultural University, Mymensingh 2202, Bangladesh; 3 Department of Genetics and Plant Breeding, Bangladesh Agricultural University, Mymensingh 2202, Bangladesh; afsana.gpb@bau.edu.bd (A.H.); naima41588@bau.edu.bd (N.S.); shirin41617@bau.edu.bd (S.A.); 4 Department of Biotechnology, Bangladesh Agricultural University, Mymensingh 2202, Bangladesh; hasanbge50450@bau.edu.bd; 5 Department of Agronomy, Bangladesh Agricultural University, Mymensingh 2202, Bangladesh; fahmida@bau.edu.bd; 6 Department of Agronomy and Haor Agriculture, Sylhet Agricultural University, Sylhet 3100, Bangladesh; sazzadmh.aha@sau.ac.bd; 7 Department of Biochemistry and Molecular Biology, Hajee Mohammad Danesh Science and Technology, Dinajpur 5200, Bangladesh; sayed_bmb@yahoo.com; 8 Faculty of Agriculture, Bangladesh Agricultural University, Mymensingh 2202, Bangladesh; toufiq47644@bau.edu.bd; 9 Department of Botany and Plant Physiology, Faculty of Agrobiology, Food and Natural Resources, Czech University of Life Sciences Prague, Kamycka 129, 165 00 Prague, Czech Republic; skalicky@af.czu.cz; 10 Northeast Institute of Geography and Agroecology, Chinese Academy of Sciences, Changchun 130102, China; lixiangnan@iga.ac.cn; 11 Institute of Plant and Environmental Sciences, Faculty of Agrobiology and Food Resources, Slovak University of Agriculture, 94976 Nitra, Slovakia

**Keywords:** abiotic stress, heavy metal, plant growth, phytomelatonin, oxidative stress

## Abstract

Heavy metal toxicity is one of the most devastating abiotic stresses. Heavy metals cause serious damage to plant growth and productivity, which is a major problem for sustainable agriculture. It adversely affects plant molecular physiology and biochemistry by generating osmotic stress, ionic imbalance, oxidative stress, membrane disorganization, cellular toxicity, and metabolic homeostasis. To improve and stimulate plant tolerance to heavy metal stress, the application of biostimulants can be an effective approach without threatening the ecosystem. Melatonin (*N*-acetyl-5-methoxytryptamine), a biostimulator, plant growth regulator, and antioxidant, promotes plant tolerance to heavy metal stress by improving redox and nutrient homeostasis, osmotic balance, and primary and secondary metabolism. It is important to perceive the complete and detailed regulatory mechanisms of exogenous and endogenous melatonin-mediated heavy metal-toxicity mitigation in plants to identify potential research gaps that should be addressed in the future. This review provides a novel insight to understand the multifunctional role of melatonin in reducing heavy metal stress and the underlying molecular mechanisms.

## 1. Introduction

Plant stresses can be classified into two categories, i.e., biotic and abiotic stress, both of which have a negative impact on plant growth, development, and yield. Living organisms, including viruses, bacteria, fungi, nematodes, insects, arachnids, and weeds, cause biotic stress [1,2]. On the other hand, abiotic stress is enforced by non-living or environmental factors [1] such as water, temperature, ultraviolet light, salt, and heavy metals (HMs) [3,4]. Abiotic stresses are associated and manifested as osmotic stress, oxidative stress, ionic imbalance, and cell metabolism dyshomeostasis, all of which influence plant growth and productivity [3,4].

Some HMs (e.g., iron (Fe), manganese (Mn), zinc (Zn), copper (Cu), molybdenum (Mo), nickel (Ni), and cobalt (Co)) are required by plants at certain concentrations, whereas when present in excessive concentrations, these elements become toxic to plants [5]. In contrast, lead (Pb), cadmium (Cd), mercury (Hg), and arsenic (As) are not required by plants, and these are highly harmful to plants [6]. The foremost feedback mechanism of plants upon exposure to elevated amounts of HMs is the production of reactive oxygen species (ROS). Most HMs cause continuous ROS production in the chloroplast, mitochondria, and peroxisomes, which can cause oxidative stress in plants and result in the unexpected consequence of HM toxicity [7,8]. HM stress frequently favors stomatal closure, increases the activity of the photorespiratory pathway, triggers the production of ROS, interrupts the antioxidant system [9], and inhibits the electron transport chain [10] as well as the plant metabolism [11]. Lipid peroxidation is a detrimental phenomenon that is triggered by HM-induced ROS, which subsequently deteriorates the cell membrane integrity and function [12,13,14].

Melatonin (*N*-acetyl-5-methoxytrytamine, MT) is a familiar pleiotropic signaling molecule that plays a role as an antioxidant by fostering plant tolerance to various biotic and abiotic stresses [15,16]. MT stimulates various physiological, morphological, and biochemical features starting from seed germination to biological yield [17,18] by the upregulation of stress-related genes [17,19] that scavenge ROS and improve the antioxidant capacity of plants against abiotic stress [16,20]. However, despite numerous research studies, the protective effect of MT against HM stress has not been thoroughly reviewed. Therefore, mitigation of HM stress by exogenous application of MT in plants is reviewed in this study, and in addition to this, we will explore the in-depth mechanisms of melatonin-mediated HM stress tolerance in plants.

## 2. Mechanism of Heavy-Metal-Induced Growth Inhibition

**Figure 1:**
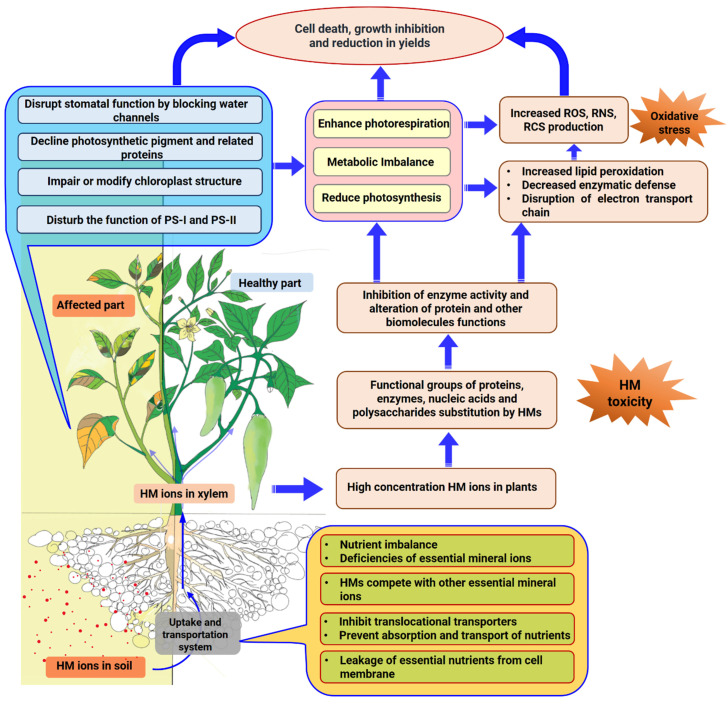
A simplified overview of the pathway demonstrating the negative effects of HMs on plants. ROS, reactive oxygen species; RNS, reactive nitrogen species; RCS, reactive carbonyl species; PS-I, photosystem-I; PS-II, photosystem-II.

Heavy metals in the growth medium have toxic effects on plants [21,22] because they cause the disruption of many key physiological processes such as photosynthesis, respiration, ROS metabolism, and hormonal regulation [23,24]. These metal elements negatively affect the life cycle of plants, from germination to final production [25]. Overall, higher concentration of HMs in plants hinders the absorption and transport mechanisms of essential nutrients, and also interrupts other metabolic processes, which affects growth, development, and yield [26,27]. The roots are the first component of a plant that detect a stress condition. As a consequence, root length and viability are reduced, which eventually disrupt the absorption of essential nutrients [28]. Because of the disruption in nutrient absorption and transport, the total chlorophyll (Chl) content of the leaves decreases, resulting in a noticeable decrease in CO_2_ assimilation rate (Figure 1) [28]. HMs also interfere with CO_2_ assimilation in plants by disrupting chloroplast ultrastructure, inhibiting absorption of light energy, disrupting the electron transport chain, and reducing stomatal conductance and activities of enzymes involved in Calvin cycle [29,30]. Under HM toxicity, photosystem II (PS II) is more affected than photosystem I (PS I) among the thylakoid components (Figure 1) [31,32]. Furthermore, as the rate of photosynthetic pigments declines, so does the productivity of the photosynthetic apparatus, leading to the photoinhibition of photosystems (Figure 1) [33]. At sub-millimolar concentrations, HMs induced stomatal closure by blocking water channels (Hg^2+^, Pb^2+^, and Zn^2+^) or ion channels (La^3+^) or downregulating tonoplast anion channels in an ABA-independent pathway. Moreover, HMs stimulate ABA accumulation in cells and ABA-induced signals might play a role in stomatal movement [34,35]. Water channels are specialized proteins that form highly selective aqueous pores across cell membranes, that are associated with water flux control and thus play a vital function in stomatal movements [36]. As a result of water channel blockage, water fluxes in guard cells are restricted, affecting stomatal movements [37].

An excessive level of HM ions in the cytosol disrupts cellular redox homeostasis and triggers oxidative stress by producing ROS [38,39], reactive nitrogen species (RNS) [40,41], and reactive carbonyl species (RCS) (Figure 1) [42,43]. Overproduction of ROS causes membrane lipid peroxidation and that leads to the formation of RCS, both of which are toxic to plant cells [42,43]. At higher endogenous levels, these ROS, RNS, and RCS are toxic and harmful to cells; cause significant damage to cellular biomolecules such as nucleic acids, proteins, and membrane lipids; and also damage the structure of chloroplasts, resulting in cell death [38,44].

Environmental stresses, including HM stress, disrupt the balance between ROS generation and detoxification by the antioxidative protection system in plants, thereby promoting oxidative stress [44]. As a result, in order to combat the overproduction of ROS, plants activate their robust antioxidant defense mechanisms [45]. Plants resist stress-induced ROS production and related adversities by directly neutralizing and removing them or indirectly by controlling the uptake, transport, translocation, and sequestration of HMs [46,47]. Enzymatic antioxidants, such as superoxide dismutase (SOD), catalase (CAT), ascorbate peroxidase (APX), glutathione peroxidase (GPX), and glutathione reductase (GR), and non-enzymatic antioxidants, such as glutathione (GSH), proline, ascorbic acid (AsA), carotenoids, and non-protein compounds rich in -SH groups, are found in plant cells [47,48,49]. Plants produce more antioxidative enzymes as a defense against oxidative damage under HM stress [49,50]. Thus, increasing the amount of antioxidants and the function of the plants’ radical scavenging systems to make them more resistant to HM stress.

Transporters are ubiquitous proteins that mediate solute translocation across cell membranes and HMs restrict the specificity of transporters in the uptake of essential elements. HM ions penetrate the cell and alter cellular and molecular functions such as metabolic pathways; starch, sucrose, and secondary metabolites biosynthesis; and ion transporter function by replacing essential ions from various binding sites of biomolecules, causing changes in the cell membrane as well as the transcriptional pattern of plants (Figure 1) [51,52]. The inclusion of HM ions to the functional groups of enzymes, proteins, and nucleic acids can disrupt metabolic processes [53]. Previous research has also shown that HM toxicity affects phosphorus (P), sulfur (S), and nitrogen (N) metabolism in addition to carbon (C) metabolism in mustard, soybean, and tomato plants [54,55,56].

Competition between HMs and nutrients occurs in various compartments of binding sites such as cell membranes and cell walls, which can influence the membrane transport system, resulting in nutrient leakage from the cell membrane [38,57]. The enzyme system serves as an interface between the cell and its surroundings for the exchange of substances and information. The stabilization of this enzyme mechanism is the foundation for the cell’s physiological functions. HM toxicity harms the enzyme mechanism and increases cell membrane penetration [58,59]. In addition to this, HMs enter into the leaves of the plant and disturb the water status of plants, which ultimately leads to osmotic stress in the plant [38]. This osmotic stress affects the growth of the plants and also causes nutrient imbalance [60,61]. Osmotic adjustment in response to stress is considered an important physiological mechanism. Plants produce and accumulate osmolytes such as soluble sugars, trehalose, amino acids, and betaines to counteract stress-induced osmotic imbalance. In addition, these osmolytes participate in cellular energy transfer, stabilize membranes and proteins, scavenge ROS, chelate HMs, and minimize metal uptake to cope with metal-induced osmotic, ionic, and oxidative stresses [62,63]. Plants with better osmoprotectant biosynthesis are adaptable to stress [64].

## 3. Heavy Metals Induce Endogenous Melatonin Accumulation in Plants

We compiled some studies here that presented the effects of HMs on the accumulation of endogenous MT in plants (Table 1). Researches showed that HMs induced endogenous MT biosynthesis in the root tissue of *Hordeum vulgare* (barley), *Solanum lycopersicum* (tomato), *Glycine max* (soybean), and *Lupinus albus* (lupin) [65,66,67,68]; leaves of *Nicotiana tabacum* (tobacco), *Arabidopsis thaliana*, and tomato [69,70,71]; and the seedlings of *Oryza sativa* (rice) [71]. Among the HMs, for example, Cd stress seriously interrupts the structural stability of cellular organelles including chloroplasts, mitochondria, and endoplasmic reticulum, resulting in the leakage of endogenous serotonin *N*-acetyltransferase (SNAT) enzymes into the cytosol [72,73], where they can easily come into contact with serotonin, resulting in *N*-acetylserotonin synthesis and subsequently MT formation [71].

Moreover, exposure to 50 µM Al^3+^ induced MT accumulation in roots and enhanced the expression of genes related to MT biosynthesis in soybean plant, which might be due to the disruption of cell membrane, whereas, in rice plants, Al^3+^ stress was unable to disrupt chloroplasts to synthesize MT even at 500 µM concentration. The concentration and exposure duration of HMs, regardless of the plant species, could be the controlling factors for endogenous MT synthesis. The content of endogenous MT increases under HM stress condition; therefore, MT might help to confer HM stress tolerance in plants.

## 4. Role of Exogenous Melatonin on Heavy Metal Stress Tolerance

Exogenous application of MT enhances plant growth attributes, such as shoot and root biomass, by alleviating the detrimental effects of HM stress. In several research articles, it has been demonstrated that the negative effects of different HMs, such as Cd, aluminum (Al), Cu, Zn, As, chromium (Cr), Cu, Fe, Pb, and Ni could be mitigated through exogenous MT applications (Table 2).

Our literature search revealed that exogenous MT improves the stress tolerance to HMs such as Al, As, Cd, Cr, Cu, Fe, Ni, and Pb of different plant species via a direct pathway, scavenging ROS directly, and via an indirect pathway by increasing antioxidant enzymes activities, photosynthetic efficiency, and metabolite content (Table 2). MT promoted the growth of *Brassica rapa* ssp. *pekinensis* (Chinese cabbage) under Al stress through improving osmotic regulation, alleviating cell membrane destruction, and protecting photosynthetic system [75]. Optimal concentrations of MT also reduce Al-induced toxic effects in soybean plants. In soybean roots, MT treatment reduced Al-induced H_2_O_2_ content by the higher activity of antioxidant enzymes such as CAT, SOD, and POD and increased citrate and malate exudation [74]. Under As stress, in rosemary herbs, exogenous foliar MT increased the contents of non-protein thiols and phytochelatins (PCs) and enhanced the accumulation of osmoregulatory substances that improved water status leading to enhanced plant growth and As stress tolerance [77]. The application of MT to rice seedlings resulted in increased plant biomass in both underground and above-ground areas. Under Cd stress, foliar MT application increased SOD and POD, while it decreased MDA in rice plant [78]. MT reversed the growth of *Medicago sativa* (alfalfa) seedlings under Cd stress by lowering Cd accumulation and restoring ROS homeostasis [67]. By lowering Cd accumulation and alleviating growth inhibition and photoinhibition, foliar application of MT improved Cd tolerance in tobacco plants [79]. MT also reduced Cd-induced oxidative damage on tobacco plants by direct scavenging of ROS by the antioxidative enzymes such as APX, CAT, and POD [79]. When apple rootstalks were treated with MT, Cd-induced decreases in development, photosynthesis, and enzyme activity were reduced, and the levels of ROS and MDA accumulation in Cd-stressed plants were reduced by MT application. Exogenous MT also regulated the mRNA levels of *HA7* (PM H^+^-ATPases 7), *NRAMP1* (natural-resistance-associated macrophage protein 1), *NRAMP3*, *HMA4* (P-type HM ATPase 4), *PCR2* (plant Cd resistance protein 2), *NAS1* (nicotianamine synthase 1), *MT2* (metallothionein 2), *ABCC1* (ATP-binding cassette transporter C1), and *MHX* (magnesium proton exchanger protein) genes involved in Cd uptake and translocation in apple plants [80]. These results suggest that MT can directly regulate the uptake and translocation of Cd in plants. External MT treatment expanded *Cucumis sativus* (cucumber) seedlings’ leaf area, ameliorated growth suppression and excessive Cd toxicity and increased photosynthetic-related parameters [81]. MT improved Cd stress tolerance in tomato plants by improving the antioxidative mechanism, increasing H^+^-ATPase activity, and sequestering Cd [68]. MT application increased fresh and dry weight while also preventing Chl content damage. It also increased the activities of antioxidant enzymes in *Spinacia oleracea* against Cd stress [83]. Cu stress reduced *Cucumis melo* (melon) growth, but when MT was applied exogenously, it improved Cu stress tolerance by enhancing redox-related gene expression and the jasmonic acid biosynthesis process [86]. Similarly, MT enhanced the tolerance of cucumber seedlings to Cu toxicity by encouraging Cu chelation and cell wall binding, controlling genes associated with ROS formation and scavenging processes, and activating enzymatic activity [87]. MT promoted new root formation in melon seedlings [86] and established essential nutrient equilibrium and triggered carbon metabolism in cucumber [87] under Cu stress. Cu stress tolerance in canola plants is conferred by MT-mediated higher levels of biomass accumulation, photosynthetic pigments, and functional efficacy of the photosynthetic apparatus, as well as lower levels of ROS, oxidative damage and Cu-induced proline accumulation [88].

MT increased tolerance to Fe-deficiency stress in *Capsicum annum* (pepper) plants by increasing active Fe content and improving K homeostasis, intracellular NO content, H_2_S content, and antioxidative enzyme activities (POD, SOD, and CAT) and decreasing H_2_O_2_ and MDA contents [89]. In cucumber, MT enhanced the internal MT level, Chl content, and CO_2_ assimilation rate, alleviated Fe-stress-induced oxidative damage, activated antioxidant enzymes, promoted secondary metabolite contents such as phenols and flavonoids, upregulated enzymes (phenylalanine ammonia lyase (PAL) and polyphenol oxidase (PPO)) involved in secondary metabolism, and altered Fe acquisition to mitigate Fe stress [90]. MT enhanced growth efficiency under Ni stress by altering the expression of MT-biosynthesis-related genes such as *TDC* (tryptophan decarboxylase), *T5H* (tryptamine 5-hydroxylase), *ASMT* (*N*-acetylserotonin methyltransferase), and *SNAT* (serotonin *N*-acetyltransferase), as well as Chl-biosynthesis-related genes such as *CHL G* (chlorophyll synthase), *POR* (cytochrome P450 Oxidoreductase), and *CAO* (chlorophyll a oxygenase), and thus mitigated the negative effects of Ni on photosynthetic pigments in tomato seedlings [91]. In tomato plants subjected to Ni stress, MT improved mineral nutrition, regulated proline, RWC, root function, and increased secondary metabolite contents such as anthocyanins, phenols, and flavonoids, as well as the antioxidant protection system [91]. MT improved Chl synthesis and plant nutrient element composition, as well as increased antioxidant defense, suppressing ROS and MDA accumulation and lowering electrolyte leakage in Pb-stressed maize and safflower plants [92,93]. Pretreatment of tobacco suspension cells with MT reduced cell death, increased cell proliferation, decreased H_2_O_2_ level, and prevented DNA fragmentation under Pb stress [94]. Moreover, MT treatment increased Bermuda grass’ resistance to Pb stress [95]. External MT treatment to *Brassica napus* (canola) enhanced growth traits under Cr stress through higher PS II efficiency and photosynthetic quotient (PQ) redox rate [85]. Application of MT in Cr-stressed *Origanum majorana* plants preserved higher levels of photosynthetic pigments and less ROS by stimulating the antioxidant machinery and osmotic balance, decreased lipid peroxidation, and improved cellular membrane integrity [77]. These results indicate that exogenous MT-mediated improvement of photosynthesis and oxidant scavenging systems enable plants to fight against Cr stress.

Therefore, research on exogenous MT revealed that it acts as an antagonist for HM toxicity, with major benefits for sustainable crop production. Exogenously applied MT helps to improve the tolerance to HM stress, but to better understand the roles of this molecule and make full use of it, many more investigations must be conducted. All these given data describe the roles of MT in plant HM stress resistance and will help to encourage plant scientists to further investigate the mechanism of MT-mediated stress tolerance.

## 5. Mechanisms of Melatonin-Mediated Heavy Metal Stress Tolerance

MT improves plants’ defense systems by modulating the antioxidant system, maintaining nutrient and metabolic homeostasis, modifying osmotic balance, stimulating secondary metabolism, increasing photosynthetic ability and controlling the expression of stress-resistant genes [96,97]. We explain the mechanistic insights of MT-mediated HM stress tolerance in plants in this section.

### 5.1. Melatonin Modulates Reactive Species Detoxification and Antioxidant Upregulation

**Figure 2:**
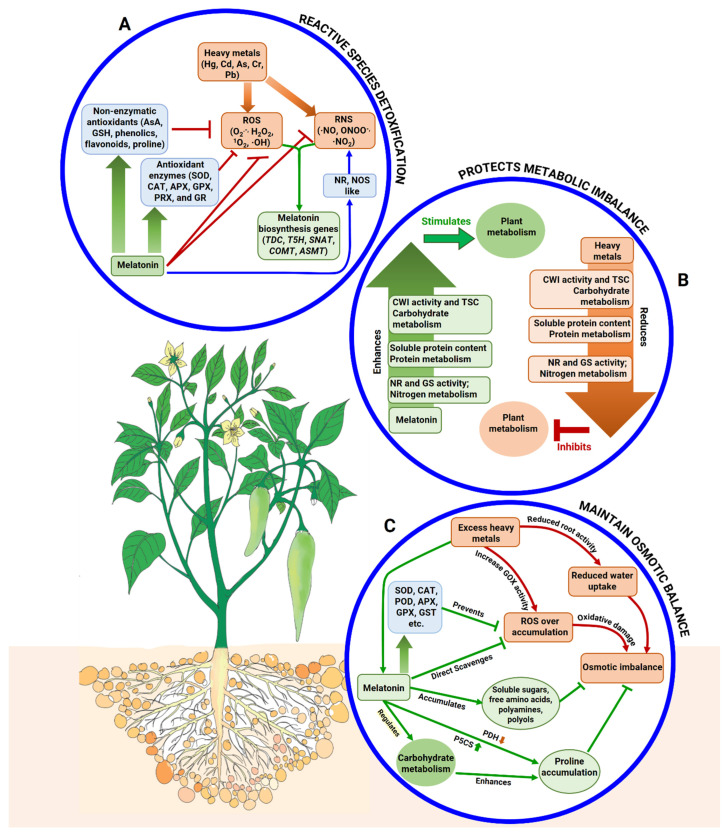
Diagram showing mechanism of MT-mediated (**A**) reactive species detoxification, (**B**) protection against metabolic imbalance, and (**C**) maintenance of osmotic balance under HM stress conditions. (**A**) ROS: reactive oxygen species, RNS: reactive nitrogen species, O_2_^•–^: superoxide anion, H_2_O_2_: hydrogen peroxide, ^1^O_2_: singlet oxygen, ^•^OH: hydroxyl radical, ^•^NO: nitric oxide, ONOO^•^: peroxynitrite, ^•^NO_2_: nitrogen dioxide, SOD: superoxide dismutase, CAT: catalase, APX: ascorbate peroxidase, GPX: glutathione peroxidase, PRX: peroxiredoxin, GR: glutathione reductase, AsA: ascorbic acid, GSH: reduced glutathione, *TDC*: tryptophan decarboxylase, *T5H*: tryptamine 5-hydroxylase, *SNAT*: serotonin *N*-acetyltransferase, *ASMT*: acetylserotonin methyltransferase, *COMT*: caffeoyl-*O*-methyltransferase, NR: nitrate reductase, NOS-like enzyme: NO synthase. **(B)** CWI: cell wall invertase, TSC: total soluble carbohydrate, GS: glutamine synthetase. **(C)** GOx: glycolate oxidase, MDA: malondialdehyde, RWC: relative water content, WSS: water-soluble sugar, P5CS: *Δ1*-pyrroline-5-carboxylate synthetase, PDH: proline dehydrogenase, POD: peroxidase.

Reactive species such as ROS, RNS, and RCS function as signaling molecules at lower concentrations. An excessive amount of free radicals such as- H_2_O_2_, O_2_^•−^, and ^•^NO is produced in plants when subjected to HM stress [98,99]. These HM-induced free radicals control lipid peroxidation and increase RCS such as MDA and cause biological membranes’ disruption [100,101]. However, oxidative stress can be alleviated by using MT, which has been extensively reported in the last few years. HM toxicity in plants can be mitigated with MT by regulating the activity of antioxidants and related gene expression such as *Cu/Zn-SOD*, *POX*, *GPX*, and *MDHAR* and by scavenging excessive reactive species (Figure 2) [102,103,104,105,106]. According to Jahan et al. [91], MT-treated tomato seedlings subjected to Ni stress upregulated the transcript levels of *PAL*, *CHS*, upregulated chlorophyll synthesis genes such as *POR*, *CAO*, and *CHL* and MT-biosynthesis-related genes such as *SNAT*, *TDC*, *T5H*, and *ASMT* (Figure 2). Positive regulation of the chlorophyll-biosynthesis-related genes and the MT-biosynthesis-related genes resulted in higher amount of endogenous MT production, which is primarily associated with scavenging excessive ROS through the enhancement of antioxidative enzyme activities (Figure 2) [105,107,108]. MT-triggered ROS scavenging and antioxidative activity upregulation were also reported in wheat, mustard, tomato, watermelon, and cucumber plants, which were observed under Cd, Pb, Fe, Zn, Cu, V, and Al stress [68,86,87,90,105,109,110,111].

Redox balance is the balance between the formation and elimination of ROS in the cellular components of plants, which is altered under stress conditions. Antioxidant systems play critical roles in reducing ROS overproduction and accumulation and the intensities of oxidative damage in plants under stress conditions [100,112,113]. MT strengthens the antioxidant activity in plants by detoxifying the excessive ROS either directly or indirectly, resulting in improved stress tolerance [114,115]. For instance, under Fe starvation and extreme conditions, application of MT can enhance the antioxidant enzymes’ activity for example, SOD, POD, and CAT, as well as their biosynthetic genes such as *Fe-SOD*, *POD*, and *CAT* exhibit increased transcription levels, indicating that MT can boost the antioxidant system in plants that functions in ROS scavenging and mitigating oxidative stress in cucumber [90]. A similar increment of ROS scavenging by means of MT application was also reported in the case of other HM stress, i.e., Cd stress in wheat [116] and strawberry [117]; and Cu stress in cucumber [87]. Furthermore, non-enzymatic antioxidants including proline, phenolics, total thiols, GSH, anthocyanin, tocopherol, and AsA were increased when plants were treated with exogenous MT [80,97,118,119]. Thus, in plants, antioxidant system efficacy can be escalated through exogenous application of MT that also vigorously scavenges ROS to improve plant tolerance to HM stress.

### 5.2. Melatonin Protects against Different Metabolic Imbalances

Synthesis of a variety of plant metabolites, especially protein, can be inhibited when HMs enter into the plant [117]. Thus, the soluble protein content of plants may be an indicator about their physiological status [120]. According to [117], under Cd stress, the soluble protein content in strawberry was higher in MT-pretreated plants than that of MT-untreated plants. This conclusion has been supported by studies on wheat and tomato subjected to Cd stress [70,116].

Soluble sugars, such as glucose, sucrose, fructose, and trehalose, perform functions as sensing and signaling molecules in plants and thereby activate or regulate several genes that are involved in defense and metabolic activities [121,122]. For example, high exposure of B (boron) on wheat seedlings had diminished the cell wall invertase (CWI) activity and levels of total soluble carbohydrates (TSC), while seedlings treated with MT had higher TSC content and CWI activity under B stress (Figure 2) [97]. The enzyme CWI is a key player in controlling the carbohydrate pathway and sugar signaling, and it is involved in a variety of metabolic roles and signaling processes in numerous plants in stressed environments [123,124]. Thus, MT emerged as a noble agent in B stress for controlling carbohydrate metabolism through enhancement of the levels of TSCs and activity of CWI (Figure 2) [97].

Moreover, N metabolism has also been linked to HMs. HMs have been found to increase protease activity and, thus, limit enzymatic activity associated with the assimilation of nitrate (nitrate reductase (NR) and nitrite reductase (NiR)) and ammonia (glutamine synthetase (GS), glutamine oxoglutarate aminotransferase (GOGAT), and glutamate dehydrogenase (GDH)). For example, nitrogen metabolism was interrupted under Cd stress as nitrate uptake and transport was inhibited through alteration of NR and GS activity [125,126,127], which ultimately influenced the processes of primary N assimilation. Exogenous MT has been reported to improve the function of NR and GS enzymes, thereby stimulating N metabolism and assimilation in plants (Figure 2) [74,118,128,129].

### 5.3. Melatonin Adjusts Osmotic Imbalance in Plants

Excessive HMs in tissues can affect water absorption from the soil by altering root morphology and anatomy, lowering the transpirational rate, blocking aquaporins, or rupturing the intercellular contacts (plasmodesmata), resulting in a decrease in water content in plants. For example, the plant–water relation was hampered by Pb stress in *Sesbania grandiflora* plants [130]. Under Ni stress, the root activity of tomato was found to be significantly reduced due to retention of water and absorption of nutrients, while pretreatment with MT sustained suitable root functioning through root repair [91]. Osmolytes are compatible solutes that increase the cell’s ability to retain water while not interfering with normal metabolism. Proline, sucrose, polyols, trehalose, glycine betaine, and polyamines are among the major osmolytes [131] that play a role to protect plants from HM stress [132] by maintaining a high water potential, turgor pressure, and water content [131]. As plant roots are the first points of contact for HM ions, they usually accumulate considerably more metal than above-ground plant parts (Figure 2) [133,134]. Proline can directly chelate HMs and, thus, reduces the effects of metals on plants [135]. MT improves proline accumulation by enhancing P5CS activity and reducing PDH activity (Figure 2) [16,136,137]. Proline accumulation also links to carbohydrate metabolism. It has been stated that proline accumulation requires carbohydrate [138]. MT increases proline accumulation by upregulating carbohydrate metabolism (Figure 2) [97]. According to [139], exogenous MT induces polyamine biosynthesis in *Arabidopsis* plants under excess-Fe and Fe-deficit conditions. Sugar accumulation is promoted by exogenous MT through protein deformation where the sulfur in S-containing proteins (Cys and Met) is substituted by Se [140,141]. Excess accumulation of free amino acids in response to MT application indicates the MT-mediated hydrolysis of protein and changes at the osmotic level (Figure 2) [140].

### 5.4. Melatonin Maintains Homeostasis of Essential Nutrients

Minerals are important in a variety of metabolic events because they sustain both water connections and plant development [128]. For plant survival, mineral absorption is important during critical physiological processes, and any changes in mineral absorption will negatively impact the plant’s metabolic activity [128]. Excessive HM accumulation in roots can cause nutrient absorption to be disrupted by root architectural distortion [142]. Under Ni-stressed conditions, for example, macronutrient contents (N, P, Mg, Ca, S) and micronutrient contents (Fe, Zn, Mn) are prominently decreased along with the reduction of root activity [91]. Since, Cd and mineral nutrients share identical pathways for transport, they have similar effects on the stability of the plasma membrane and balance at an ionic level [143].

**Figure 3:**
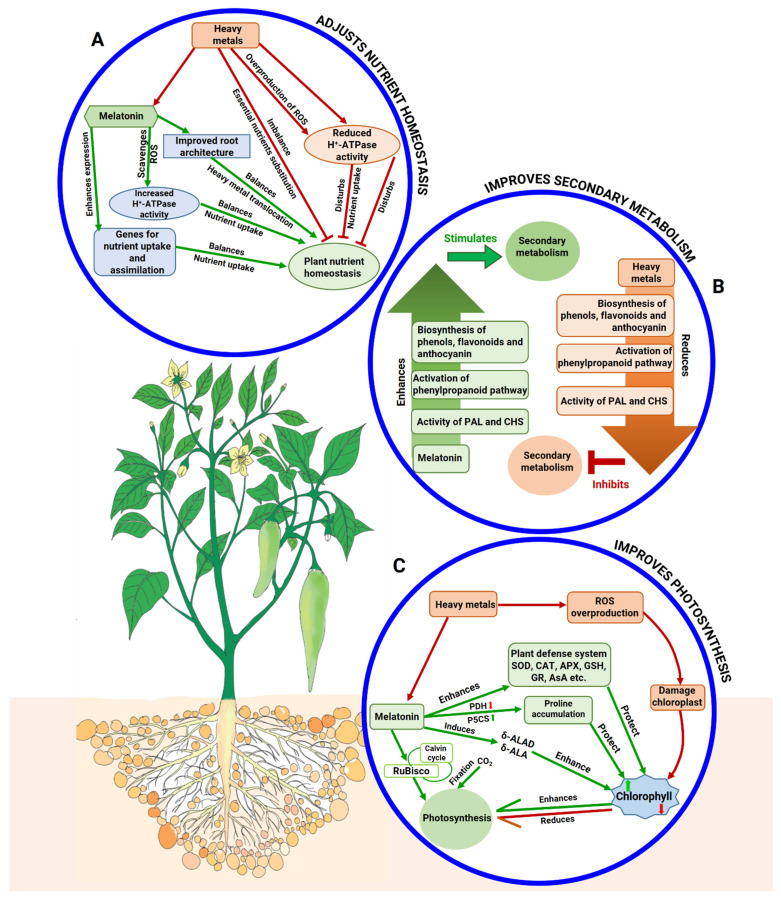
Schematic diagram showing MT-mediated (**A**) maintenance of nutrient homeostasis, (**B**) improvement in secondary metabolism, (**C**) enhancement in photosynthesis in plants under different HM stresses. (**A**) ROS: reactive oxygen species, H^+^-ATPase: proton pump ATPase. (**B**) PAL: phenylalanine ammonia-lyase, CHS: chalcone synthase. (**C**) GOx: glycolate oxidase, CO_2_: carbon dioxide, Chl: chlorophyll, CA: carbonic anhydrase, δ-ALA: δ-aminolevulinic acid and δ-ALAD: δ-aminolevulinic acid dehydratase, Pro: proline, P5CS: Δ1-pyrroline-5-carboxylate synthetase activity, PDH: proline dehydrogenase, SOD: superoxide dismutase, CAT: catalase, APX: ascorbate peroxidase, GSH: reduced glutathione, GR: glutathione reductase, AsA: ascorbic acid.

MT significantly affects the plant nutrient composition and alleviates HM stress by maintaining the balance of nutrient elements probably by improving the root architecture of plants (Figure 3). In tomato, MT might play a key role in nutrient homeostasis maintenance [91]. MT application recovered the negative effects of Ni, which remarkably increased N, P, Mn, and Mg concentrations in tomato leaves and roots [91]. MT treatment improves the architecture of roots via inhibiting embryonic root growth, stimulating lateral root formation and activating auxin-related genes synthesis and the exudation of organic acid anion, which might prevent the translocation and accumulation of HMs [74,105,106]. The transmembrane electrochemical proton gradient is an important factor in the nutrient uptake process where the activity of the plasma-membrane-based H^+^-ATPase can be impaired by higher ROS generation from HM toxicity (Figure 3) [144]. MT can be converted into 5-methoxytryptamine, and thus, it enhances H^+^-ATPase activity in plants (Figure 3) [145]. MT can also increase the uptake of different nutrients by enhancing gene expression. For instance, MT caused overexpression of the caffeoyl-O-methyltransferase (*COMT*) gene that enhances sulfate transporter *SUT1* and *SUT2* genes, that improved S-uptake and assimilation in tomato plants [146]. Likewise, in *Arabidopsis* plants, MT increased Fe uptake by enhancing the expression of Fe acquisition genes, such as ferric reductase-oxidase2 (*FRO2*) and iron-regulated transporter1 (*IRT1*) [139].

### 5.5. Melatonin Regulates Secondary Metabolites

In plants, a large number of compounds are provided through secondary metabolism that mainly function to enhance plant tolerance to diverse stressors [68,147]. Secondary metabolites, for example, anthocyanins, phenols, and flavonoids, specifically participate in combating HM stress by chelating metals (Fe^2+^ and Cd), restricting the synthesis of free radicals and reducing the ROS level [96,148,149]. For instance, anthocyanins hinder •OH formation via chelating Fe^2+^ [150]. HM concentration is considered as a crucial parameter that affects the response of plants in secondary metabolism production. Lower levels of HMs enhance the production of secondary metabolites; on the contrary, higher concentrations inhibit the synthesis of secondary metabolites in plants [151]. Fe-toxicity significantly affected the expression of genes such as *chalcone isomerase* in rice roots that are associated with the biosynthesis of flavonoids and phenolics [152]. MT regulates secondary metabolite production by enhancing the activity of enzymes phenylalanine ammonia-lyase, (PAL), chalcone-synthase (CHS), and dihydroflavonol-reductase (DFR) responsible for the biosynthesis of secondary metabolites (Figure 3). Numerous secondary metabolites are produced from the phenylpropanoid pathway; a key biosynthetic route for secondary metabolites synthesis, in which PAL acts as the premier line rate-limiting enzyme [90,153]. Ahammed et al. [90] reported that application of MT considerably increased the activity of PAL together with increasing the concentrations of phenols and flavonoids under Fe stress in cucumber plants. Jahan et al. [91] also found that the concentration of phenols and flavonoids was enhanced by MT treatment in Ni-stressed tomato seedlings. Similar findings were observed in pepper plants under B [154] and rosemary herb under As [77] toxicity supported by the attributes of MT (Figure 3).

The MT-deficient *Arabidopsis* mutant accumulated low levels of anthocyanin [96,155]. However, the addition of MT improved the anthocyanin content in tomato plants under Ni stress [91] and in rosemary herb under Cr stress. Under non-stress condition, MT treatment significantly improved the levels of expression of different anthocyanin-biosynthesis-related genes such as *PAL*, cinnamic acid 4-hydroxylase *(C4H)*, chalcone synthase *(CHS)*, chalcone isomerase *(CHI)*, flavanone 3-hydroxylase *(F3H)*, flavonoid 3′-hydroxylase *(F3′H)*, dihydroflavonol 4-reductase *(DFR)*, and leucoanthocyanidin dioxygenase *(LDOX)* in cabbage plants [96]. The transcript level of CHS is enhanced in MT-treated pepper plants that are exposed to Ni stress, demonstrating that MT might participate in regulating the anthocyanin concentration and in mitigating Ni and As toxicity [77,154].

### 5.6. Melatonin Protects Photosynthetic Attributes

The plant photosynthesis system is interrupted due to HM toxicity by decreasing Chl biosynthesis, carbonic anhydrase (CA) activity, weakened cell wall expansion, cell division and accumulation of lignin and suberin in plants [156]. Jahan et al. [97] reported that excessive mineral stress increased Chl degradation and the activity of Chl degrading enzyme (Chlase) and lessened the universal biosynthetic precursor compound (δ-ALA) of the photosynthetic pigments and the activity of δ-ALAD in wheat seedlings (Figure 3). Moreover, excessive HM causes oxidative damage, and this may lead to the injury of reaction centers, modify thylakoids structure, and induce irregular growth of soft parenchymatic tissue [21,120].

Exogenous MT improves the photosynthesis rate under HM toxicity by stimulating enzymes engaged in the photosynthetic pathway and pigment biosynthesis (Figure 3). As for example, foliar application of MT amplified biosynthesis of photosynthetic pigments by lessening Chl degradation by the downregulation of Chlase activity and increasing the activity of δ- ALAD, CA, RuBisco, and the content of δ-ALA [97]. MT can lessen the Chl degradation rate and upsurge Chl content to progress plant photosynthesis in Cd stress [104,157]. These positive contributions of MT in the reestablishment of altered Chl synthesis and enzyme activity might be traced based on its role in the biosynthesis of porphyrins, glycine, and succinyl-CoA by regulating the activity of δ-aminolevulinate synthase [158]. MT also increases ferredoxins that prevent Chl from degradation. Ferredoxins reduce the overproduction of high-energy electrons from the photosynthetic electron-transport chain and raise the level of reduced AsA and diminish ROS levels that eventually protect Chl from degradation [159]. Augmented activity of carbonic anhydrase (CA) and RuBisco in MT-treated tomato seedlings under B toxicity may have enhanced carbon fixation by conserving acid–base balance, ion exchange, and also continuous supply of CO_2_ [16,160], which was reproduced in improving photosynthesis rate. MT treatment robustly increases the photosynthesis efficiency in watermelon [105] and tomato [82] under vanadium (V) stress by increasing the photosynthesis and antioxidant enzyme activities and, finally, delaying leaf senescence. Furthermore, in tomato plants, the PS II (Fv/Fm) smooth functioning has been observed by MT application under stress combinations [161].

### 5.7. Melatonin Upregulates Defensive Genes

HMs activate diverse signaling pathways in plants, for example, calcium-dependent (CDPKs) signaling, mitogen-activated protein kinase signaling (MAPKs), ROS signaling, and hormone signaling [162,163]. MT modifies the expression of genes that participate in the signal transduction phases along the way.

MT is a signaling molecule that can upsurge gene expression or activities of antioxidant enzymes in HM stress [74,118]. For instance, the relative expression of antioxidative genes for SOD, APX, and GPX was upregulated in MT-treated watermelon seedlings exposed to V stress [105]. Plant HM toxicity can also be repelled by stimulating the biosynthesis of metal-binding peptides, such as phytochelatins (PCs) [164]. Application of MT and GSH in safflower seedlings under Zn stress, increased the PC content, which could be partially related to the enhancing role of MT in encouraging transcription of the genes engaged in encoding the enzymes accountable for the biosynthesis PCs [165]. MT upregulated several metal transporter genes, including *ZIP12* (zinc-iron permease 12), *HMA4* (heavy metal ATPase 4), *YSL2* (yellow stripe-like transporter 2), and *YSL7* (yellow stripe-like transporter 7) subjected to long-distance transport of Cd and stimulated the transport of Cd beyond the radish root cell and *CAX4* (vacuolar cation/proton exchanger 4), ATP-binding cassette (ABC) transporters (*ABCC14*, *ABCB21*, *ABCG39*) responsible for sequestration of Cd into the vacuole [166]. MT also increases photosynthetic efficiency via increasing Chl biosynthesis gene expression. For example, the relative expression of Chl biosynthesis genes, i.e., *CHL G*, *POR*, and *CAO* genes, were expressed in MT-treated seedlings under Ni stress [91]. Similar experimental results were conveyed by [105], who specified that MT increases the Chl content via modifying the transcript level of Chl biosynthetic gene in watermelon under V stress. The introduction of these genes, which is crucial in HM stress signaling in the presence of MT, indicates the composite cross-talk between MT and HM stress response.

## 6. Endogenous MT in HM Stress Tolerance

Overexpression of the *HsfA1a* gene triggers synthesis of endogenous MT in tomato plants through the increased expression of the MT biosynthetic gene *COMT1* [70]. Eventually, *HsfA1a* overexpression confers Cd stress tolerance by triggering endogenous MT that upsurges antioxidant capacity [70]. Moreover, overexpression of the *AtASMT* gene in rice plants increases endogenous MT biosynthesis in the cytoplasm, which in turn enhances Cd tolerance via antioxidant and metal chelate formation [69]. Another research’s findings in rice plants concluded that rice mutants overexpressing *ASMT*, *SNAT1*, and *SNAT2* genes improve MT biosynthesis, which also conferred Cd tolerance [71]. Thus, several studies demonstrated that higher endogenous MT accumulation can enhance HM stress tolerance in plants. However, many researchers have focused on overexpressing MT for controlling other abiotic stresses, but so far very few studies have been reported on HM stress. Therefore, further experiments should be conducted to reveal the putative mechanisms of endogenous MT-mediated HM stress tolerance. Furthermore, we found no research concentrating on the response of MT-deficient mutants to HM stress; consequently, further investigations into the detailed responses of MT-deficient mutants to HM stress condition are required.

## 7. Conclusions and Future Prospective

The purpose of this review is to keep readers up to date on the role of MT in HM stress mitigation and to encourage plant scientists to dig deeper into the mechanism of MT-mediated tolerance. Exogenous MT acts as a potent plant growth regulator that improves the overall growth and productivity of plants under HM stress.

- Exogenous MT significantly improves the concentration of photosynthetic pigments by upregulating Chl synthesizing enzymes and downregulating genes responsible for Chl degradation.
- Exogenous MT alleviates the harsh effects of HM stress on plant growth, photosynthesis, and development.
- Exogenous MT mitigates HMs toxicity through upregulating a wide range of defensive genes that are responsible for higher antioxidant activities and metal chelating properties.
- The application of MT enhances HM tolerance in plants by the accumulation of osmolytes, increased antioxidant enzyme activity, and osmotic adjustment, maintaining membrane integrity and limiting the lipid peroxidation and ROS generation, improving the activity of antioxidant enzymes and non-enzymatic antioxidants.
- MT also improves different nutrient homeostasis.
- The exogenous application of MT induces plant secondary metabolites biosynthesis.

Despite the fact that MT has piqued the interest of plant biologists and some progress was made in recent times, the complicated signaling pathways controlled by MT under HM stress conditions are still relatively unexplored. There is indeed a lack of understanding about the genes and main pathways that MT regulates precisely. Furthermore, a number of basic problems must be addressed. The mechanism of MT-regulated HM uptake, transportation, and sequestration is still not fully understood. Many further studies are needed to better understand the functions of this molecule and to ensure sustainable use of those mechanisms to ensure better crop production under HM stress conditions.

## Figures and Tables

**Table 1:** Effects of HMs on the levels of endogenous melatonin accumulation in plants.

HMs	Plant Species	Exposed Organs	Concentration (µM)	Exposure Duration	MT Level	Reference
Al	*Glycine max*	Root	50	24 h	↑	[74]
Al	*Nicotiana tabacum*	Leaves	100	15 days	↑	[71]
Al	*Oryza sativa*	Seedlings	500	72 h	↓	[71]
Cd	*Medicago sativa*	Root	50, 100, and 200	15 h	↑	[67]
Cd	*Solanum lycopersicum*	Root	100	15 days	↑	[68]
Cd	*Solanum lycopersicum*	Leaves	100	15 days	↑	[70]
Cd	*Nicotiana tabacum*	Leaves	500	15 days	↑	[71]
Cd	*Arabidopsis thaliana*	Leaves	300	24 h	↑	[69]
Cd	*Oryza sativa*	Seedlings	500	72 h	↑	[71]
Zn	*Hordeum vulgare*	Root	100	15 h	↑	[65]
Zn	*Hordeum vulgare*	Root	100	72 h	↑	[65]
Zn	*Lupinus albus*	Root	100	24 h	↑	[66]

↑ (increase), ↓ (decrease), h (hour).

**Table 2:** Role of exogenous melatonin in plants exposed to various HM stress.

Plant Species	HM (conc.)	MT Doses	Observed Effects of MT on Plant Systems		Reference
			Increase	Decrease	
*Brassica rapa*	Al (50 µM)	50, 100, 200, and 400 µmol L^−1^	SOD, POD, and CAT activity; SP and Chl content; RWC; plant growth and biomass.	MDA content.	[75]
*Glycine max*		0.1, 1, 10, 100, and 200 mM	CAT, SOD, and POD activity; exudation of malate and citrate; gene encoding *acetyltransferase* NSI-like (nuclear shuttle protein-interacting); root growth.	H_2_O_2_ content.	[74]
*Oryza sativa*	As (25 µM)	250 mM	GSH, PCs content; dry matter production.	Oxidative stress; H_2_O_2_ and MDA content.	[76]
*Rosmarinus officinalis*	As (75 mg kg^−1^ soil)	25, 50, 100, and 200 µM	SOD, POD, and CAT secondary metabolites (AsA, phenols, flavanoids); chloroplast ultrastructure; Chl pigment, essential ions, essential oil, stability, the cell membrane integrity; growth.	H_2_O_2_ and MDA content.	[77]
*Oryza sativa*	Cd (10 and 50 µM)	10 and 50 µM	SOD and POD activity; plant biomass including both underground and above-ground areas.	Accumulation of Cd; transcription of Cd uptake and transport-related genes.	[78]
*Medicago sativa*	Cd (50, 100, and 200 µM)	10, 50, and 200 µM	Cd tolerance; microRNA-mediated redox homeostasis.	Accumulation of Cd and ROS; oxidative damage.	[67]
*Nicotiana tabacum*	Cd (10, 50, 100, and 200 µM)	25, 50, 100, and 250 µM	APX, CAT, and POD content; promotion of cell wall or vacuolar sequestration of Cd; plant growth.	Expression of Cd uptake-related genes (*IRT1*, *Nramp1*, *HMA2*, *HMA4*, and *HMA3*); photoinhibition.	[79]
*Malus micromalus*	Cd (30 µM)	0 and 100 µM	Photosynthesis, photosynthetic pigments; transcriptionally regulated key genes involved in detoxification; plant biomass.	Cd-induced reductions in growth; ROS and MDA.	[80]
*Cucumis sativus*	Cd (100 µM)	150 µmol L^−1^	LA; photosynthetic rate; Chl content; stomatal conductance; transpiration rate.	Growth inhibition; excess Cd poisoning.	[81]
*Solanum lycopersicum*		100 µM	APX, POD, and CAT activity; redox homeostasis; S metabolism, and biosynthesis of downstream S metabolites; H^+^-ATPase activity; GSH and PCs; plant growth.	Oxidative stress.	[68]
*Cyphomandra betacea*	Cd (10 mg L^−1^)	50 µM	SOD, POD, and CAT activity; biomass of *C. betacea* seedlings; Cd contents in the stems, leaves, and shoots of *C. betacea* seedlings.	Seedlings growth inhibition	[82]
*Spinacia oleracea*	Cd-As (25, 75, and 125 ppm)	100 µM	SOD, POD, and CAT activity; fresh and dry weight.	Chl damage; lipid peroxidation.	[83]
*Melissa officinalis* and *Valeriana officinalis*	Zn-Cd (3 g L^−1^ and 15 mg L^−1^)	1 µM	POD and CAT activity; SP content.	MDA content; oxidative stress.	[84]
*Brassica napus*	Cr (50 and 100 µM)	0, 1, 5, and 10 µM	SOD, POD, APX, and CAT activity; photosynthesis rate; photosystem II efficiency; cellular redox potential; plant growth and development.	Cr accumulation; ROS accumulation.	[85]
*Cucumis melo*	Cu (300 µM)	10, 50, 100, 300, 500, and 800 µmol L^−1^	SOD, POD, and CAT activity; GSH which chelates excess Cu^2+^; redox-related gene expression; cell-wall-related gene expression.	ROS production.	[86]
*Cucumis sativus*	Cu (80 µM)	10 nmol L^−1^	SOD, APX, POD, and GR activity; GSH and PC content; Cu^2+^ sequestration; carbon metabolism (glycolysis and the pentose phosphate pathway); cell wall trapping; plant fresh weight.	Cu^2+^ toxicity and ROS production.	[87]
*Brassica napus*	Cu (10–100 µM)	0.1–100 µM	Plant biomass; photosynthetic pigments; efficiency of photosynthetic apparatus; proline content.	Oxidative stress.	[88]
*Capsicum annum*	Fe (0.1 mM)	100 µM	POD, SOD, and CAT activity; Chl content; active Fe^2+^ and K^+^ content; endogenous NO and H_2_S; total biomass; fruit yield of plants.	H_2_O_2_ and MDA content.	[89]
*Cucumis sativus*	Fe (3 and 90 mg L^−1^)	100 µM	Endogenous MT content; SOD, POD, and CAT activity; phenols and flavonoids contents; phenylalanine ammonia lyase, polyphenol oxidase activity; photosynthetic pigment and rate; plant growth and biomass.	ROS production and Fe acquisition.	[90]
*Solanum lycopersicum*	Ni (50 µM)	100 µM	APX, CAT, SOD, POD and GR activity; redox balance; Chl-synthesis-related genes; photosynthesis rate; phenols, flavonoids, and anthocyanin content; nutrient homeostasis; biomass production.	ROS accumulation.	[91]
*Zea mays*	Pb (0.1 mM)	0.05 and 0.10 mM	SOD, POD, and CAT activity; nutrient element content; plant growth.	Oxidative stress; H_2_O_2_ and MDA content; electrolyte leakage.	[92]
*Carthamus tinctorius*	Pb (50 µM)	0–300 µM	APX, CAT, SOD, and POD activity; glyoxalase (Gly I and Gly II); Chl and PC content; biomass production of roots, stems and leaves.	Pb stress.	[93]
*Nicotiana tabacum*	Pb (15 µM)	200 nM	Pb stress tolerance.	Programmed cell death; ROS content; DNA fragmentation.	[94]
*Cynodon dactylon*	Pb (1000 and 2000 mg kg^−1^ soil)	0, 5, 20, and 100 µM	SOD, CAT, POD, APX, and GR activity; non-enzymatic antioxidant (AsA and GSH) content; water status; photosynthetic pigments; biomass production.	ROS content; membrane lipid peroxidation and permeability.	[95]

SOD, superoxide dismutase; CAT, catalase; POD, peroxidase; APX, ascorbate peroxidase, GR, glutathione reductase; GSH, glutathione; GSSG, oxidized glutathione; SP, soluble protein; RWC, relative water content; LA, leaf area; PC, phytochelatin, AsA, ascorbic acid; LOX, lipoxygenase; TPC, total phenolic compounds; NPT, non-protein thiols; MDA, malondialdehyde; TSS, total soluble sugars.

## Data Availability

Not applicable.
